# Long-term follow-up after discharge witnesses a slow decline of insulin autoantibodies in patients with insulin autoimmune syndrome complicated with Grave’s disease: a report of two cases

**DOI:** 10.1186/s12902-023-01410-6

**Published:** 2023-08-17

**Authors:** Lili Zhao, Jinzhi He, Shandong Ye, Chao Chen, Jie Zhu, Chunchun Xiao, Tingni Wu, Zhicheng Liu

**Affiliations:** 1 Department of Endocrinology, The First Affiliated Hospital of USTC, Division of Life Sciences and Medicine, University of Science and Technology of China, Hefei, Anhui 230001 China; 2 School of Pharmacy, Anhui Medical University, Hefei, Anhui 230032 China

**Keywords:** Insulin autoimmune syndrome, Grave’s disease, Hypoglycemia, Iodine therapy, Genotype test

## Abstract

**Background:**

Insulin autoimmune syndrome (IAS) is a rare cause of hypoglycemia characterized by high levels of blood insulin autoantibodies. It has been documented that drugs containing sulfhydryl groups may result in IAS. In this study, we present two cases of IAS induced by methimazole, along with their corresponding treatments and a long-term follow-up after hospitalization.

**Case presentation:**

We report two patients with Grave’s disease (GD), carrying the HLA-DRB1 04:06 genotype, who experienced hypoglycemic episodes after taking methimazole. Inpatient treatments helped return their blood glucose levels to normal. Although no recurrences of hypoglycemia were present in the two cases studied, insulin autoantibodies remained positive for the previous follow-up sessions, which turned negative only three years after discharge.

**Conclusions:**

GD patients who carry the HLA-DRB1 04:06 genotype are prone to IAS if they take drugs containing sulfhydryl groups. It may take time for the elimination of insulin autoantibodies after the recovery from the hypoglycemic episode in IAS patients.

## Introduction

Insulin autoimmune syndrome (IAS), also known as “Hirata’s disease,” is a rare condition characterized by spontaneous hypoglycemia accompanied by the presence of insulin autoantibodies (IAA) in the blood, either fasting or postprandial [1]. IAS was first reported in Japan in the 1970s. To date, the mechanism behind the occurrence of IAS remains unclear. It is speculated that IAS is induced by the dysregulations of T lymphocytes. IAS is usually complicated by other autoimmune diseases, such as Grave’s disease (GD) and rheumatoid arthritis [2].

Moreover, people who have been exposed to drugs containing sulfhydryl groups, such as methimazole are prone to IAS [3]. This side effect is related to the reducing activity of sulfhydryl-containing compounds, which dissociate the S-S bound in insulin and expose the alpha chain of insulin to the antigen-presenting cells [4]. Regarding the genotypic characteristics of IAS patients, the presence of HLA-DR4 alleles, including HLA-DRB1*0403, HLA-DRB1*0406, and HLA-DRB1*0407, have been previously documented [5]. Therefore, while the assay of insulin autoantibodies in the blood remains the first line of diagnosis, genotype analysis helps confirm the diagnosis of IAS.

IAS is usually considered a self-remission condition with a good outcome. However, the therapy remains challenging, as no therapeutic guideline has been put forth until now, and IAS patients often have other comorbidities. Moreover, recurrences of hypoglycemia are common after treatment, and a long follow-up period is recommended. Nonetheless, according to previous studies, few follow-ups of IAS patients have been conducted for more than one year [6, 7]. In this study, we report on two cases of IAS with GD induced by methimazole therapy. we also performed a three-year follow-up on the patients to better understand their recovery after discharge.

## Case presentations

### Case 1

A 39-year-old Chinese woman with a BMI of 22.64 kg/m^2^ was admitted to a regional hospital due to loss of consciousness, sweating, palpitation, and intense hunger feelings. The symptoms occurred after physical labor and during a bath. Convulsion and incontinence were absent. The symptoms improved after glucose infusion. Similar symptoms reappeared ten days later, and the patient was transferred to our department. The blood glucose level was 2.4 mmol/L (43.2 mg/dl) during hospitalization and 1.4 mmol/L (25.2 mg/dl) during another hypoglycemic event ten days after admission.

The patient did not have a history of diabetes or hypoglycemia, did not smoke or drink alcohol, did not take anti-diabetic drugs or insulin, and had grade II thyroid enlargement without eye protrusion. She was diagnosed with Graves’ disease (GD) ten months before hospitalization and was taking 15 mg of methimazole and 30 mg of propranolol per day before the first hypoglycemia event. At admission, her blood pressure was 134 over 78 mm/Hg, and her heart rate was 90 bpm. She experienced recurrent palpitations and sweating at night and in the morning.

Table 1 shows the patient’s blood glucose, insulin, and C-peptide levels and oral glucose tolerance test (OGTT) during two hypoglycemic events experienced during hospitalization, which indicated that the hypoglycemia events were related to increased blood insulin. The glycosylated hemoglobin (HbA1c) levels were 6.2%. The patient’s blood insulin antibody (IAA) was tested with an Iodine[^125^I] Insulin Antibody Assay Kit(Binding Assay, North Institute of Biotechnology Co, Ltd.)according to the manufacturer’s instructions. Positive IAA was found in the patient (IAA = 19.18%, reference: IAA < 5%). No abnormalities in cortisol, adrenocorticotropic hormone, catecholamine, and growth hormone were found. A computed tomography (CT) examination on pancrease did not report abnormality. The patient had diffuse thyroid enlargement, and no abnormal changes were observed in the pancreas. HLA-DNA typing identified DRB1*0406 for the patient, confirming the diagnosis of IAS.

**Table 1:** Hypoglycemia-associated indicators measured for case 1 during hospitalization. Reference value: average blood glucose: 4.2–8.8 mmol/L (75.6–158.4 mg/dl) Insulin: 15.28–176.63 mmol/L; C-peptide: 0.33–1.60 nmol/L.

Measurement	During hypoglycemia events		OGTT Test					
	First episode	Second episode	Fasting	30 min	60 min	120 min	180 min	240 min
Glucose (mmol/L)	1.71	1.05	4.12	9.44	10.2	15.2	11.6	12.3
Insulin (pmol/L)	1427.51	1486.23	1843.67	1594.76	1704.09	1802.27	1844.8	1641.5
C-peptide (nmol/L)	3.84	3.67	4.57	4.4	4.47	4.46	4.97	4.57

The patient was treated with dextrose drip therapy to restore her blood glucose level during hypoglycemic episodes, but the effect was not satisfactory. She was then treated with 30 mg/d of prednisone during her hospital stay, which she withdrew herself twenty days after discharge. She was discharged 19 days after admission, and the lowest fasting blood glucose (FBG) level was 3.5 mmol/L (63 mg/dl) during the last five days before discharge. Multiple small meals a day and a low-carbohydrate diet were prescribed to prevent the recurrence of hypoglycemic events. Radio ablation iodine therapy was performed for the care of GD instead of methimazole.

**Fig. 1:**
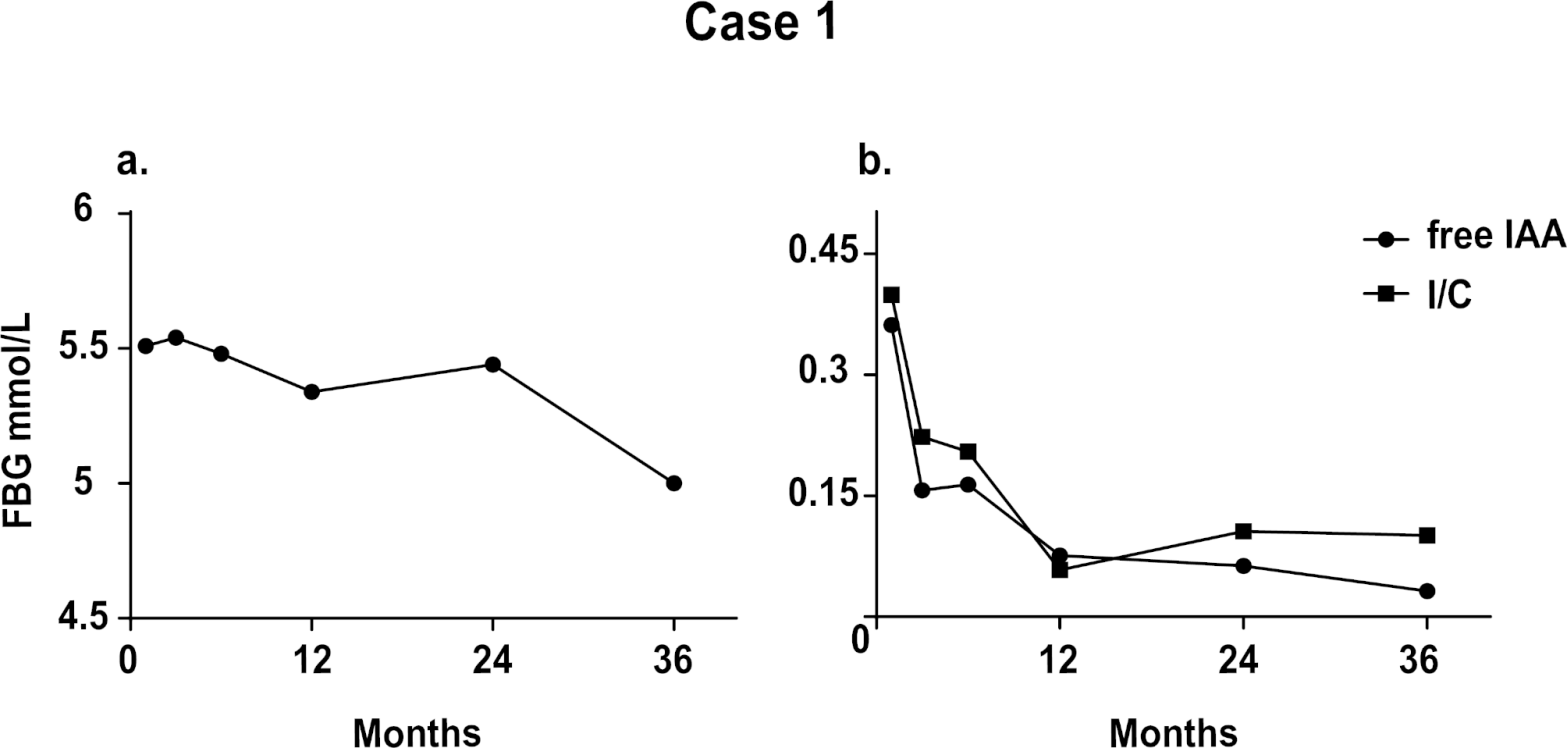
IAS-associated indicators measured along with the three-year following-up in Case 1. a. Fasting blood glucose; b: IAA and the ratio of insulin on C-peptide (I/C) measured for the sessions of follow-up

The patient was followed up three years after discharge, and no recurrence of hypoglycemic events was reported. Blood glucose was generally stable during the follow-up, as shown in Fig. 1a. A progressive decrease in IAA was observed in the patient, and IAA turned negative until the last follow-up visit (Fig. 1b). The ratio of insulin on C-peptide decreased with time, which was consistent with the decrease in IAA. During the follow-up, the patient experienced hypothyroidism three months after the ablation treatment, as indicated by the test of thyroid-associated indexes T3, T4, and TSH. Levothyroxine sodium was prescribed to balance the secretion of thyroid hormone.

### Case 2

The second case report involves a 50-year-old woman (BMI = 27.47 kg/m^2^) who had previously been diagnosed with hyperthyroidism eight months prior to the first occurrence of hypoglycemia. She underwent thiamazole therapy (10 mg/day for seven months) in a regional hospital. She was preliminarily diagnosed with hypoglycemia upon admission to the hospital when she experienced consciousness loss for the first time (blood glucose (BG) level = 2.0 mmol/L (36 mg/dl)). She also experienced hunger and limb weakness during the hypoglycemia event. The patient was transferred to our department due to repeated episodes of consciousness loss, palpitation, and sweating without any apparent cause. Her blood pressure at hospitalization was measured at 134/93 mmHg, and her heart rate was 82 bpm.

The patient had a history of hypertension and GD, for which she was taking felodipine for blood pressure control. The patient did not have diabetes or exposure to anti-diabetic drugs. She was a non-smoker and did not consume alcohol before hospitalization. No noticeable abnormalities were found during the electrocardiogram and imaging examinations.

The patient experienced two episodes of consciousness loss during hospitalization when her BG levels were lower than 2.8 mmol/L (50.4 mg/dl). The level of HbA1c was 5.5%. Table 2 shows the results of the OGTT test for the hypoglycemia event. Like case 1, the patient exhibited enhanced blood insulin during the hypoglycemia attacks. IAA was measured in the patient, by the same way as it was done in case 1, and it was found to be at 28.6%. There were no abnormalities with cortisol, adrenocorticotropic hormone, catecholamines, and growth hormone tests. No abnormalities were found in the patient’s pancrease according to a CT examination. An investigation of genotype showed that the patient carried HLA-DRB1*0406.

**Table 2:** Associated indexes measured for the hypoglycemia event occurred during hospitalization in case 2

Measurement	During the hypoglycemia event	OGTT Test					
		Fasting	30 min	60 min	120 min	180 min	240 min
Glucose (mmol/L)	2.7	4.63	9.58	13.17	12.19	8.1	0.93
Insulin (pmol/L)	705.51	726.98	1319.08	1907.27	9669.3	10,279	2026.3
C-peptide (nmol/L)	1.44	1.72	2.86	3.22	3.99	4.11	2.41

The patient was treated with an intravenous glucose drip to ameliorate the hypoglycemia events without any other drug therapy. A series of radioactive thyroid therapy was conducted during hospitalization to treat GD. The patient was advised to consume small but frequent meals with low carbohydrates per day. She was discharged ten days after admission. No hypoglycemia episodes were experienced from the last five days before discharge until the present.

**Fig. 2:**
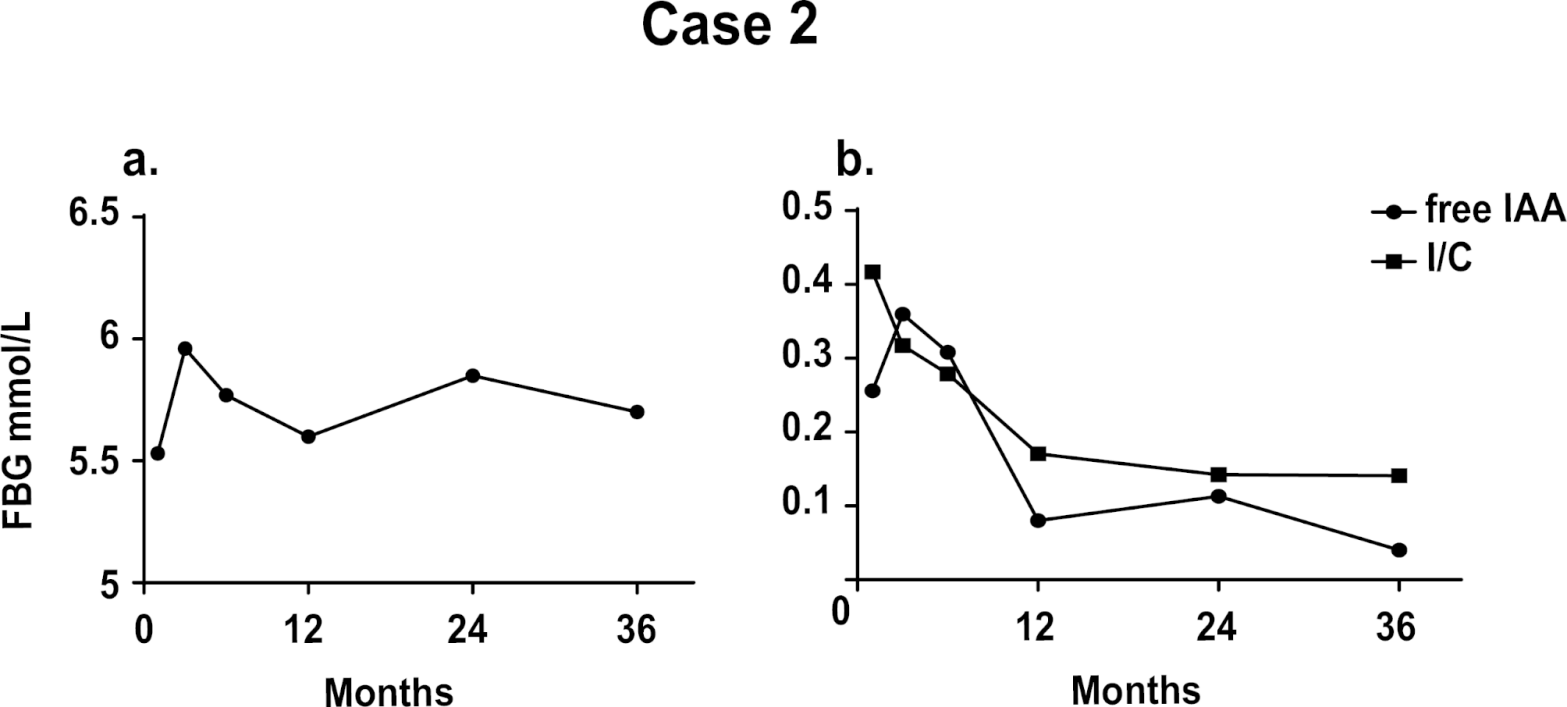
IAS-associated indicators measured along with the three-year following-up in Case 2. a. Fasting blood glucose; b: IAA and the ratio of insulin on C-peptide (I/C) measured for the sessions of follow-up

Similar to case 1, this patient was followed up for three years. Unfortunately, there is missing data of the follow-up 3 months after discharge due to the patient’s absence. The blood levels of fasting glucose, IAA, insulin, and C-peptide tested during the follow-up sessions are shown in Fig. 2a-b. Correspondingly, the IAA of the patient was found to be decreasing and became negative up to the last visit. The insulin/C-peptide ratio was consistent with the gradual decline of IAA since the second visit three months after her discharge. However, her hypothyroidism did not improve as expected at the one-year follow-up after discharge. Increased dosages of levothyroxine sodium were suggested, but the patient did not comply. After the latest follow-up, the patient was advised to take 100 µg/d to recover from radioiodine-induced hypothyroidism.

## Discussion

IAS is a rare but important cause of hypoglycemia. Differential diagnosis is needed to avoid misdiagnoses (e.g. diabetes, insulinoma) and prevent neural injuries resulting from severe hypoglycemic episodes. The BG tests in both fasting and postprandial periods help to identify the hypersecretion of insulin during the early stage of diabetes. The radiographs are also indicated when the patient suffers from recurrent hypoglycemia, to avoid the misdiagnosis of insulinoma. Moreover, knowledge of autoimmune comorbidities and drug exposure history, as well as IAA and insulin levels in the patients are crucial for early diagnosis of IAS.

Here we report two typical cases of IAS complicated with GD. The patients were initially diagnosed with hypoglycemia and were therefore pre-treated with glucose supplementation before administration into our department. Their IAS were diagnosed according to further work-up such as IAA measurement and genotypic investigation. While we investigated the patients’ medical records, we realized that the occurrence of IAS was related to exposure to sulfhydryl group-containing drugs. Particularly, the second case had been taking methimazole for 8 months before she developed IAS. The development of other autoimmune polyglandular syndromes (APS) in the patient was considered but was ruled out by imaging and hormone analyses. This case was rare since most works reported that the onset of IAS-induced hypoglycemia was about 2–6 weeks after the administration of the drug [8–10]. However, notably, we find that one case who developed IAS 3 months after taking methimazole was reported by Torimoto and co-workers [11]. Given that high levels of IAA in IAS patients are associated with the dissociation of the insulin-IAA complex, the delayed onset of IAS was speculated to be due to the individual stability of the insulin-IAA complex. Notably, alternated hypo- and hyperglycemia can be possibly observed in IAS patient. As to the OGTT data, abnormally enhanced BG was found 120 min after glucose intake in the two patients. Such dysregulations of BG are supposed to be due to the binding between insulin and IAA, which decreases blood insulin and tiggers increased BG in the patients. The sulfhydryl group-containing drugs used for treating GD were thereafter replaced by radioiodine therapy. Our genetic tests revealed that the patients’ human leukocyte antigens (HLA) were both DRB*04:06. Thus, our findings confirm the evidence that HLA-DRB1*0406 is associated with IAS susceptibility in Asians. Rather than HLA-DRB1*0403 or HLA-DRB1*047, which are documented as genetic predispositions for IAS in Caucasians, the presence of HLA-DRB1*0406 is more frequently present in Asian IAS patients [12]. Besides, it has been recognized that the mutations of HLA-DRB are closely related to the expression of IAA [13]. Hence, people with the above genotypes are prone to IAS with exposure to sulfhydryl-containing drugs. For the ones carrying aforementioned genotypes, IAA and insulin tests should be recommend prior to the administration of SH-containing drug.

Despite favorable outcomes for most IAS cases, a long-term following-up is considered significant since recurrent hypoglycemia may be present after hospitalization. As proof, high levels of IAA were recorded in both two cases, especially in the first year after the discharge. IAA for the two cases turned negative until their last visit although declines in IAA were observed during the three-year follow-ups. In addition, the insulin/C-peptide ratios for the two patients were in parallel with the variation of IAA according to the follow-up measurements (Figs. 1b and 2b). Growing data have documented that the insulin/C-peptide ratio is of potential value for the diagnosis of IAS [14], which is confirmed by our findings. Besides, our data from the follow-up manifest the normal BG levels in the patients three years after discharge, proving that the hypoglycemic episodes and postprandial hyperglycemia during their hospital stay were not triggered by diabetes.

Our literature investigation revealed that only one study by Yoshino and his colleagues conducted a longer follow-up in an IAS patient than we did. IAA in the reported case decreased gradually along with a continuous therapy of prednisolone [15]. By contrast, our data suggest that the decline of IAA during the follow-up might be spontaneous, even though the two cases reported herein did not take any drug for maintaining BG after their discharge.

## Conclusion

In conclusion, IAS induced by methimazole therapy is a rare but important complication in patients with GD who carry the genotype HLA-DRB1 04:06. The diagnosis of IAS is mainly based on the measurement of insulin autoantibodies in the blood, and the genotypic analysis of IAS patients can help confirm the diagnosis. Inpatient treatments can help to return the blood glucose levels to normal, but recurrences of hypoglycemia are common after the treatments. A long follow-up is necessary for IAS patients, even if the hypoglycemia-like symptoms are relieved, as it may take time for the elimination of insulin autoantibodies after the recovery from the hypoglycemic episode.

## Data Availability

The main data have been shown in the manuscript. Other data used and/or analyzed during the current study are available from the corresponding author upon reasonable request.

## Abbreviations

IAS: Insulin autoimmune syndrome
GD: Grave’s disease
IAA: Insulin autoantibodies
OGTT: Oral glucose tolerance test
HbA1c: Glycosylated hemoglobin
FBG: Fasting blood glucose
CT: Computed tomography
