# Correction: Long-term follow-up after discharge witnesses a slow decline of insulin autoantibodies in patients with insulin autoimmune syndrome complicated with grave’s disease: a report of two cases

**DOI:** 10.1186/s12902-023-01505-0

**Published:** 2023-11-10

**Authors:** Lili Zhao, Jinzhi He, Shandong Ye, Chao Chen, Jie Zhu, Chunchun Xiao, Tingni Wu, Zhicheng Liu

**Affiliations:** 1 Department of Endocrinology, The First Affiliated Hospital of USTC, Division of Life Sciences and Medicine, University of Science and Technology of China, Hefei, 230001 Anhui China; 2 School of Pharmacy, Anhui Medical University, Hefei, 230032 Anhui China

**Correction:** ***BMC Endocr Disord*** **23, 177 (2023)**

10.1186/s12902-023-01410-6

Following publication of the original article [1], the authors reported the order of “Division of Life Sciences and Medicine” and “The First Affiliated Hospital of USTC” has been reversed in the published affiliation 1.

The correct order of affiliation 1 should read:

Department of Endocrinology, The First Affiliated Hospital of USTC, Division of Life Sciences and Medicine, University of Science and Technology of China, Hefei, Anhui, 230,001, China.

The original article [1] has been updated.
